# Global Pharmaceutical Regulation: Comparative Frameworks and Operations

**DOI:** 10.3390/pharmacy14020050

**Published:** 2026-03-18

**Authors:** Omolayo Tinuke Umaru, Adebowale Sylvester Adeyemi, Olajumoke Aderonmu, Balyodh Singh Bhangu, Harjot Singh Dhaliwal, Hae Lim, Taiwo Opeyemi Aremu

**Affiliations:** 1 Department of Pharmaceutical Care & Health Systems (PCHS), College of Pharmacy, University of Minnesota, 308 Harvard Street SE, Minneapolis, MN 55455, USA; umaru002@umn.edu (O.T.U.); adeye040@umn.edu (A.S.A.); 2 Department of Public Health, Western Illinois University, 1 University Circle Road, Macomb, IL 61455, USA; oi-aderonmu@wiu.edu; 3 Department of Clinical & Social Pharmaceutical Sciences, College of Pharmacy, Touro University, 1310 Club Dr., Vallejo, CA 94592, USA; bbhangu@student.touro.edu (B.S.B.); hdhaliwa3@student.touro.edu (H.S.D.); 4 Cardiac Ablation Solutions (CAS), Medtronic, 8200 Coral Sea Street NE, Mounds View, MN 55112, USA; hae.w.lim@medtronic.com; 5 Cardiac Rhythm Management (CRM), Medtronic, 8200 Coral Sea Street NE, Mounds View, MN 55112, USA; 6 Division of Environmental Health Sciences, School of Public Health, University of Minnesota, 420 Delaware Street SE, Minneapolis, MN 55455, USA

**Keywords:** pharmacovigilance, drug development, post-marketing surveillance, global pharmaceutical regulation, regulatory frameworks

## Abstract

Pharmaceutical regulation plays a central role in protecting public health by governing clinical trials, market authorization, and post-marketing safety monitoring throughout the medicine life cycle. While substantial literature describes established systems, particularly the United States Food and Drug Administration (FDA), Japan’s Pharmaceuticals and Medical Devices Agency (PMDA), and the European medicines regulatory network coordinated by the European Medicines Agency (EMA) together with national competent authorities, comparative analyses that integrate both mature authorities, emerging regulators and transnational harmonization networks remain limited. This narrative review draws on primary regulator/network documentation and targeted peer-reviewed literature to compare core regulatory functions across jurisdictions, including approval pathways and evidentiary expectations, inspection and good manufacturing practice (GMP) oversight, transparency practices, and pharmacovigilance and risk-management approaches. Across regions, we observe increasing convergence in scientific expectations through initiatives such as the International Council for Harmonisation (ICH) and reliance and work-sharing models, alongside persistent differences in legal mandates, resourcing, timelines, and data requirements. These differences are most consequential for complex products (e.g., advanced therapies) and in crisis settings, where emergency or conditional authorizations amplify the need for strong lifecycle monitoring, real-world evidence governance, and cross-border communication. We conclude by outlining opportunities to strengthen regulatory resilience and equity through fit-for-purpose harmonization, investment in enabling infrastructure, and future work on interoperable data systems, signal detection, and coordinated post-marketing evaluation.

## 1. Introduction

The pharmaceutical industry operates within an increasingly complex and interconnected regulatory environment designed to ensure the safety, efficacy, and quality of medicinal products worldwide. Regulatory affairs (RA) is a key function of the pharmaceutical industry, overseeing the development, approval, manufacturing, and ongoing monitoring or post-marketing surveillance of pharmaceutical agents to ensure they meet ever-evolving regulatory standards. As healthcare challenges become more global in nature and medical innovations advance at an unprecedented pace, regulatory affairs play an essential role in ensuring consistency, stability, and compliance with international best practices and standards. Regulatory frameworks must therefore continue to evolve while maintaining their fundamental role in protecting public health [1].

The last decade has witnessed significant transformations in regulatory approaches, driven by technological advancements, emerging health threats, and the growing need for international collaboration [2]. These changes include the adoption of expedited review pathways, reliance models, the integration of real-world evidence, and increased emphasis on post-marketing surveillance systems capable of proactive risk detection. The COVID-19 pandemic and the emergence of the COVID-19 vaccines revealed the importance of cross-border regulatory collaboration, catalyzing joint reviews, emergency use authorizations, and digital innovation in regulatory operations.

Beyond compliance, regulatory affairs professionals facilitate drug approvals by navigating complex submission processes, addressing regulatory requirements across different regions, and ensuring timely market entry. They also serve to align global dossiers across regions to meet the essential needs of medical humanity. Furthermore, post-marketing surveillance mechanisms overseen by regulatory affairs (RAs) help identify safety signals, enabling proactive risk management and ongoing product life cycle oversight. International collaboration amongst regulatory authorities across countries brings several advantages, including better access to affordable medications, the exchange of expertise, alignment of regulatory standards, and initiatives to strengthen regulatory capabilities [3].

While the literature is replete with studies of the United States Food and Drug Administration (FDA) [4,5,6,7], the European Medicines Agency (EMA) [6,7,8], and Japan’s Pharmaceuticals and Medical Devices Agency (PMDA) [3,9], this paper examines the critical roles of regulatory affairs in pharmaceutical development and marketing, emphasizing the importance of international collaboration and harmonization in an increasingly interconnected global medical ecosystem. It also compares both established and emerging regulatory authorities across regions and explores how different systems address these challenges while accounting for regional requirements and population heterogeneity. This review focuses on human medicinal products (drugs and biologics, including vaccines and advanced therapies) and compares regulators and regulatory networks at the level of their core pharmaceutical functions. It does not cover medical device regulation or veterinary/nonhuman medicines; other regulated product categories (e.g., cosmetics) are mentioned only where needed to explain agency mandates or terminology. Key comparative information is condensed into three summary tables to support the synthesis.

### Review Approach and Sources

This article is a structured narrative review that compares key elements of pharmaceutical regulation for human medicines across major jurisdictions and regulatory networks. The objective is to synthesize and critically interpret differences and commonalities in (i) product authorization pathways, (ii) clinical trial authorization and oversight, (iii) manufacturing quality/good manufacturing practice (GMP) expectations and inspections, and (iv) post-marketing pharmacovigilance and risk management.

To develop the comparative tables and narrative synthesis, we used two complementary evidence streams: (1) primary sources (e.g., official websites and published guidance from national medicines regulators and regulatory networks; relevant legislation and regulatory manuals when available) and (2) secondary sources from the peer-reviewed literature (review articles, policy analyses, and empirical studies relevant to regulatory processes and performance). Peer-reviewed literature was identified through targeted searches of bibliographic databases and scholarly search engines, using combinations of keywords related to pharmaceutical regulation, marketing authorization, GMP, inspections, pharmacovigilance, and the specific regulator/network names (e.g., FDA, EMA, PMDA, and World Health Organization), with additional snowballing from reference lists and citation tracking of key sources. Priority was given to current regulatory guidance and to the most recent peer-reviewed analyses when describing pathways, terminology, and harmonization initiatives.

Information included in the summary tables was cross-checked against primary regulator/network sources where possible. Where inconsistencies were identified between secondary literature and primary sources, the tables and text were revised to reflect the most current and authoritative primary documentation available. Because regulatory frameworks evolve, the review emphasizes stable structural features while noting selected recent reforms where directly relevant.

## 2. Global Regulatory Landscape

### 2.1. Global Regulatory Authorities

Global markets have undergone significant evolution in recent decades, characterized by an unprecedented expansion of cross-border trade and investment activities, including healthcare. This heightened interdependence among nations underscores the necessity for robust regulatory frameworks to manage risks and promote international relations effectively. Regulatory authorities play a central role in these systems, ensuring safety, uniformity and stability, and compliance across jurisdictions.

#### 2.1.1. Established Major Regulatory Authorities

Among several other agencies, the FDA, PMDA, and the European medicines regulatory network—coordinated through the EMA in partnership with national competent authorities (NCAs) in European Union/European Economic Area (EU/EEA) Member States—constitute the principal regulatory systems governing clinical trials, market authorization, and post-marketing surveillance worldwide. These systems, encompassing the United States, Europe, and Japan, maintain distinctive regulatory frameworks that shape the landscape of pharmaceutical drug development and approval processes. Together, they represent some of the most comprehensive pharmaceutical regulatory approaches, with highly structured review processes and international impact [10].

1. The U.S. Food and Drug Administration (FDA)

The FDA, one of the oldest regulatory bodies, was established to address safety concerns surrounding medical supplies and patent medicines, leading to the enforcement of the Pure Food and Drug Act of 1906 [11]. Initially, its authority was limited to preventing the adulteration and misbranding of food and drugs, as outlined before the Federal Food, Drug, and Cosmetic (FD&C Act) of 1938 [12]. As an example, thalidomide-related birth defects prompted the introduction of the Kefauver–Harris Amendments, which mandated pre-market approval and required evidence of both safety and effectiveness [13]. These reforms shaped the pre-market regulatory framework still in use today. The FDA enforces laws and guidelines that form the basis of its regulatory authority, primarily outlined in the FD&C Act [14]. The FDA is responsible for protecting public health by ensuring the safety and efficacy of drugs, medical devices, food, cosmetics, and tobacco products, advancing medical innovations, providing science-based information to the public, and supporting national counterterrorism efforts [15,16].

2. Europe’s medicines regulatory network: the European Medicines Agency (EMA) and national competent authorities (NCAs)

Medicines regulation in Europe is delivered through a coordinated network of the EMA, the European Commission, and NCAs in EU/EEA Member States. Established in 1995, EMA fosters scientific excellence in the evaluation and supervision of medicines and supports timely patient access to innovative therapies through its adaptive pathways initiative [17,18]. In the centralized procedure, the EMA is responsible for the scientific evaluation of marketing authorization applications; once granted by the European Commission, the centralized marketing authorization is valid in all EU Member States as well as Iceland, Norway, and Liechtenstein [19]. NCAs are primarily responsible for authorizing medicines that do not fall under the scope of the centralized procedure and may also be responsible for other activities related to medicinal products, including authorization of clinical trials [20,21]. In pharmacovigilance, EU law requires marketing authorization holders, NCAs, and EMA to operate pharmacovigilance systems, with overall safety monitoring operating through cooperation between Member States, EMA, and the European Commission [22].

Finland’s Finnish Medicines Agency (Fimea) provides an example of a national authority within the EU network, combining regulation, medicines information, and pharmacovigilance. Fimea regulates and develops the pharmaceutical sector and promotes the rational use of medicines by producing and providing drug information, while also overseeing pharmacovigilance nationally as part of the EU’s agency network [17,23]. Finland’s Kanta Prescription Centre stores all electronic prescriptions and associated dispensing notes in a central service, supporting medication safety and enabling comprehensive analyses of national prescription drug utilization [24,25].

Similarly, Sweden’s Medical Products Agency (Läkemedelsverket) is the national authority responsible for the regulation and surveillance of the development, manufacturing, and sale of medicinal products [26]. Sweden also maintains a National Prescribed Drug Register, established in 2005, which contains all prescribed drugs dispensed in pharmacies and provides a strong foundation for official statistics and real-world drug utilization research [27].

3. Japan’s Pharmaceuticals and Medical Devices Agency (PMDA)

The PMDA is a Japanese regulatory agency governed by the Ministry of Health, Labour, and Welfare, recognized for its role in protecting public health by assuring the safety, efficacy and quality of pharmaceuticals and medical devices [28]. This agency was established and became functional on 1 April 2004, under the Law for the Pharmaceuticals and Medical Devices Agency, as a consolidation of the services of the Pharmaceuticals and Medical Devices Evaluation Center of the National Institute of Health Sciences (PMDEC), the Organization for Pharmaceutical Safety and Research (OPSR/KIKO), and parts of the Japan Association for the Advancement of Medical Equipment (JAAME) [29].

#### 2.1.2. Emerging Major Regulatory Authorities

The BRIC countries (Brazil, Russia, India, and China) featured below constitute major emerging pharmaceutical markets distinguished by their large, fast-growing economies and significant regional influence, positioning them as increasingly important regulatory powers in the global pharmaceutical landscape [30]. In general, these agencies perform comparable core functions in pharmaceutical oversight, although their legal authorities, evidentiary expectations, and operational maturity differ from those of the FDA, PMDA, and the EU regulatory network.

1. China’s National Medical Products Administration (NMPA)

The National Medical Products Administration (NMPA), previously known as the China Food and Drug Administration (CFDA), has its origins in the State Food and Drug Administration (SFDA). In March 2013, the SFDA was renamed and restructured as the CFDA, achieving the status of a ministerial-level agency [31]. Subsequently, as part of China’s governmental reorganization, the NMPA experienced another transformation in March 2018, which involved its consolidation into the newly established State Administration for Market Regulation (SAMR) [31]. The National Medical Products Administration (NMPA) is responsible for regulating the safety, quality, registration, and post-marketing management of drugs, medical devices, and cosmetics, while also formulating policies, standards, and laws, overseeing professional qualifications, and engaging in international cooperation in these areas [31].

2. Brazil’s National Health Surveillance Agency (ANVISA)

The Brazilian Health Surveillance Agency (Agencia Nacional de Vigilancia Sanitaria—ANVISA) was established in January 1999 [32,33]. Its primary objective is to protect public health by managing risks associated with health-related goods and services. ANVISA’s main focus includes maintaining scientific standards and regulating and inspecting products that may endanger health. Operating under the Brazilian National Health System (SUS), ANVISA is responsible for overseeing the production of health goods and healthcare services in both the public and private sectors [32,33]. Its duties include enforcing industry regulations, inspecting manufacturing practices, and registering and monitoring health products and procedures. Furthermore, ANVISA plays a significant role in the implementation of healthcare policies, such as pharmaceutical assistance, and in directing scientific research related to product development [32,33].

3. India’s Central Drugs Standard Control Organization (CDSCO)

The Central Drugs Standard Control Organization (CDSCO) serves as the central authority under the Drugs and Cosmetics Act, overseeing the approval of new drugs, clinical trials, drug standards, and quality control of imported drugs and coordinating state drug control activities [34,35]. The Drug Controller General of India (DCGI) is responsible for approving licenses for specified categories of drugs such as blood and blood products, intravenous (IV) fluids, vaccines, and sera [34]. CDSCO’s mission is to safeguard public health in India by ensuring the safety, efficacy, and quality of drugs, cosmetics, and medical devices [34].

4. Russia’s Federal Service for Surveillance in Healthcare (Roszdravnadzor)

The Federal Service for Surveillance in Healthcare (Roszdravnadzor) was established by the President of the Russian Federation through Decree No. 314 dated 9 March 2004, “On the System and Structure of the Federal Executive Bodies”, and serves as a federal executive body responsible for overseeing and regulating the healthcare system [36]. It also protects public health by ensuring safety and quality of medicines and medical devices manufactured in or imported into the Russian Federation.

#### 2.1.3. Other Regulatory Authorities

While the FDA, the European medicines regulatory network (EMA and national competent authorities), PMDA, and emerging BRIC authorities command significant influence on the pharmaceutical landscape, other regulatory bodies also contribute essential regional perspectives and represent important pharmaceutical markets in their respective regions. Apart from Brazil’s ANVISA, Argentina’s ANMAT provides complementary South American representation, as it follows a distinct regulatory approach and serves the second-largest pharmaceutical market in the region. This dual representation from South America mirrors our approach to Europe and North America, where the EU medicines regulatory network and the FDA provide established reference frameworks for large regional markets. The following regulatory authorities are therefore from regions not previously addressed, ensuring that all inhabited continents are represented in this global regulatory review.

1. Argentina’s National Administration of Drugs, Food and Medical Technology (ANMAT)

ANMAT (Administración Nacional de Medicamentos, Alimentos y Tecnología Médica) of Argentina represents a key regulatory authority in South America, having been awarded the status of “Regulatory Authority of Regional Reference” by the Pan American Health Organization and gaining recognition as an ICH observer member in 2019 [37]. ANMAT’s responsibilities extend beyond pharmaceuticals to include food safety, medical technology evaluation, and registration requirements for imported medical products, operating within a comprehensive regulatory framework that serves as a benchmark for neighboring countries [38].

2. Australia’s Therapeutic Goods Administration (TGA)

Australia’s Therapeutic Goods Administration (TGA) stands as the preeminent regulatory authority for Oceania, with its influence extending throughout the Asia-Pacific region [39]. It also served as the 2022 chair of the International Medical Device Regulators Forum (IMDRF) and participated in international work-sharing initiatives with other major global regulatory authorities [39]. The TGA’s core activities include the regulation of medicines, medical devices, blood and blood products, and vaping products and advertising; however, it does not regulate food, dietary supplements or veterinary drugs. The agency also oversees import and export controls to ensure consistent quality standards across Australia and neighboring Pacific countries [39].

3. Selected African Regulatory Agencies (NAFDAC, SAHPRA, TMDA, EDA, Ghana’s FDA, MCAZ, ARP, and Rwanda’s FDA)

The African Medicines Regulatory Harmonization (AMRH) initiative, established in 2009, aims to facilitate and coordinate the harmonization of medicines regulation through Regional Economic Communities (RECs) and improve access to quality, safe, efficacious, and affordable medicines in Africa [40]. Alongside this, the WHO benchmark, conducted with its Global Benchmarking Tool, evaluates regulatory systems against more than 250 indicators. Maturity Level 4, the highest level, signifies an advanced regulatory system committed to ongoing improvement. Maturity Level 3 indicates a stable, well-functioning, and integrated regulatory system [41]. Currently, out of 54 African countries, only eight have achieved WHO Maturity Level 3 certification for their national medicines regulatory authorities, [41] including

- Nigeria (NAFDAC)—National Agency for Food and Drug Administration and Control;
- South Africa (SAHPRA)—South African Health Products Regulatory Authority;
- Tanzania (TMDA)—Tanzania Medicines and Medical Devices Authority;
- Egypt (EDA)—Egyptian Drug Authority;
- Ghana (FDA)—Food and Drugs Authority;
- Zimbabwe (MCAZ)—Medicines Control Authority of Zimbabwe;
- Senegal (ARP)—Agence Sénégalaise de Réglementation Pharmaceutique;
- Rwanda (FDA)—Food and Drugs Authority.

##### Nigeria’s NAFDAC

NAFDAC merits focus as Nigeria’s pharmaceutical market represents the largest in West Africa, accounting for 60% of the region’s market [42]. NAFDAC stands out among the eight WHO Maturity Level 3 certified regulatory authorities in Africa for its leadership in regional pharmaceutical regulation and for overcoming severe challenges with counterfeit medications [43]. Another notable success was the pioneering of the Mobile Authentication Service (MAS), which allows consumers to verify product authenticity via short message service, otherwise known as SMS [44,45]. NAFDAC has also appointed accredited laboratories in China and India to effect the pre-shipment analysis and certification of drugs and pharmaceutical supplies coming into Nigeria [43]. Overall, the agency regulates and controls the importation, exportation, manufacture, advertisement, distribution, sale and use of food, drugs, cosmetics, medical devices, bottled water, chemicals and detergents [46].

### 2.2. Harmonization Initiatives and Comparative Regulatory Frameworks

#### 2.2.1. Global Harmonization Efforts

Despite advances in local regulatory systems, there remains a strong demand for global harmonization of science-based standards to streamline drug development and evaluation and to improve product quality, safety, and efficacy worldwide. In recent years, initiatives to harmonize application requirements across jurisdictions have aimed to minimize redundant clinical trials, reduce costs, address disparities in safety monitoring and access to healthcare, and facilitate drug distribution while maintaining safety and efficacy.

The International Council for Harmonisation of Technical Requirements of Pharmaceuticals for Human Use (ICH) was established in 1990 [47]. It was designed to promote harmonization so that safe, effective, and high-quality medicines are developed, registered, and maintained in the most resource-efficient manner while meeting high standards [47]. ICH was initiated by regulatory authorities and industry associations from Europe, Japan, and the United States [47,48]. In its current governance structure, the ICH Management Committee includes representatives of the six founding members (European Commission, the U.S. FDA, Japan’s Ministry of Health, Labor and Welfare (MHLW), European Federation of Pharmaceutical Industries and Associations (EFPIA), Pharmaceutical Research and Manufacturers of America (PhRMA), and the Japan Pharmaceutical Manufacturers Association (JPMA)), along with standing regulatory members such as Health Canada and Swissmedic and observers including the WHO and the International Federation of Pharmaceutical Manufacturers and Associations (IFPMA) [48,49]. This harmonization has reduced the duplication of clinical trials and procedures, lowering bureaucracy and shortening the time to market for new drugs [50].

#### 2.2.2. Regional Harmonization Networks

Nations around the world often participate in a variety of regional harmonization networks that support and reinforce the global harmonization efforts. Regional harmonization can increase the efficiency of regulatory authorities by drawing from a shared pool of resources and expertise. Less-resourced National Regulatory Authorities (NRAs) see harmonization as a way to enhance collaboration, strengthen decision-making, share resources, reduce duplication, and improve public health outcomes [50].

### 2.3. Comparative Analysis of Regulatory Authorities

Regulatory systems vary considerably in their legal mandates, operational frameworks, review models, and degree of alignment with international standards. To characterize these differences systematically, Table 1 provides a comparative overview of core regulatory features across a set of established (FDA, EMA, and PMDA) and emerging (NMPA, ANVISA, CDSCO, and Roszdravnadzor) agencies. For a detailed version including twenty feature comparisons and explanatory descriptions, see Table S1. The features assessed span pre- and post-market regulatory requirements, scientific and procedural standards, and engagement with global harmonization initiatives, thereby offering a multidimensional perspective on the evolving global regulatory landscape.

## 3. Drug Development and Approval Process

### 3.1. Clinical Trial Oversight

#### 3.1.1. Global Clinical Trial Standards

As a crucial process in drug development, the role of clinical trials cannot be overstated. Clinical trials ensure novel drugs are rigorously tested for efficacy and safety while adhering to ethical standards. Global frameworks such as the Belmont Report have become foundational practices held by clinical researchers and pharmaceutical industries when conducting studies involving human subjects to protect study participants [62]. Other important global standards include the International Ethical Guidelines for Biomedical Research Involving Human Subjects, issued by the Council for International Organizations of Medical Sciences [63] and the International Council for Harmonisation (ICH), and the Guideline for Good Clinical Practice (GCP). These guidelines are globally recognized and are upheld as a tool for guiding clinical trial processes for new medicines [64,65].

#### 3.1.2. Innovation in Clinical Trial Regulation

Innovative approaches are reshaping clinical trial regulations to enhance efficiency and adaptability. A recent study by Rosa et al. suggested leveraging real-world data collected through digital technologies for improving clinical trial processes [66]. The study also highlighted the absence of sufficient regulatory frameworks to guide the use of digital tools in clinical trials [66]. The authors also emphasized the need to ensure the quality and representativeness of data potentially sourced from participants’ electronic records, thereby simplifying trial design and data collection. The advancement and growing need for innovative approaches in clinical trial design calls for adapting regulatory frameworks to support data quality and responsible methodological flexibility. For example, the U.S. Food and Drug Administration’s Center for Drug Evaluation and Research (CDER) recently established the Center for Clinical Trials Innovation (C3TI) to enable and amplify innovative clinical trial approaches intended to improve the efficiency of drug development [67].

Furthermore, a study by Scheppler et al. identified the role regulatory authorities play in accelerating global access to life-saving vaccines and advocated for harmonization and collaboration of national regulatory agencies in clinical trial processes [68]. Collaboration among regulatory agencies is key to helping solve the lengthy drug approval challenge and ultimately to ensuring that patients receive the safe and effective treatments they deserve.

In Sub-Saharan Africa, a recently ratified collaboration shows the interest of African regulatory agencies in working toward well-regulated clinical trials through the African Medicines Agency (AMA) [69]. Hwenda et al. explored the paucity of clinical trial data in Africa—which accounts for less than 3% of global clinical trial genomics data and the readiness of the AMA to accelerate drug development and improve clinical trials in Africa [69]. Africa has untapped potential to contribute clinical trial data given its 1.3 billion population, diverse populations and disease burden [70]. As a regulatory initiative, the AMA is well positioned to improve the continent’s contribution to clinical development.

### 3.2. Review and Approval Mechanisms

Drug review and approval mechanisms vary considerably between countries. In the U.S., the FDA relies on preclinical and clinical trial data from drug development to make an approval decision for a New Drug Application (NDA). Each NDA undergoes a thorough review that typically lasts 6 to 10 months to verify the efficacy, safety and adequacy of the development program. A decision for approval is made when the review committee determines that the new drug is safe and effective for its intended use. FDA review processes can also be expedited through accelerated approval and priority review pathways, which allow faster evaluations of therapies addressing serious or life-threatening conditions [11,71].

### 3.3. Challenges in Multinational Clinical Trial Regulations and Potential Solutions

Conducting multinational clinical trials presents significant challenges, as these trials must comply with diverse local and international laws, regulations, and guidelines that differ from one country to another. The incompatibility of these regulatory frameworks, combined with a lack of adequately trained Good Clinical Practice (GCP) staff at clinical sites and difficulties in ensuring effective oversight across different jurisdictions, can result in delays in study timelines and increased costs.

One potential solution to enhance global collaboration in clinical trials is the creation of a more unified regulatory framework that offers all trial sites a common reference structure for regulatory review and informed consent processes. Such a system would enable national regulators to collaborate on approvals, despite differences in political, social, and economic contexts, while maintaining decentralized oversight within each country. Additionally, establishing a centralized support network could provide mentorship to less experienced researchers and facilitate the exchange of expertise and technology throughout the trial process, addressing challenges related to limited professional expertise and infrastructure.

## 4. Post-Market Safety Monitoring

### 4.1. Pharmacovigilance Systems

Pharmacovigilance (PV) systems are an extension of the original adverse drug reaction (ADR) monitoring and reporting systems and are also internationally recognized systems that must align with the entire drug life cycle and post-marketing supervision of drugs.

#### Internationally Recognized Pharmacovigilance (PV) Systems

Various internationally recognized drug safety surveillance frameworks exist worldwide: the EU monitoring network, the WHO-Uppsala Monitoring Centre approach, and the ICH framework. Despite differences in operational structures and implementation, they all aim to promote safe medication use [72]. The ten pioneering members of the Uppsala Monitoring Centre include Australia, Canada, the Federal Republic of Germany, Ireland, the Netherlands, New Zealand, Sweden, the United Kingdom, the United States and former Czechoslovakia. These nations were the first to respond to the thalidomide tragedy by developing an international adverse drug reaction reporting network [71].

The WHO’s global initiative for medication safety surveillance, known as the Programme for International Drug Monitoring (WHO-PIDM), is a global network of participating countries and territories with varying levels of pharmacovigilance capacity. Because PV systems require significant financial and technical resources, low- and middle-income countries (LMICs) often struggle with budgetary and workforce constraints, leading to limited or non-functional PV systems. In contrast, high-income countries are often better positioned to sustain mature PV activities, although network participation should not be conflated with equivalent operational capacity [71].

### 4.2. Risk Management and Mitigation

Across the globe, regulatory authorities have prioritized risk assessment as well as risk mitigation strategies [73]. These approaches help ensure continued product safety while maintaining market access.

#### 4.2.1. Risk-Management Plan (RMP)

One key risk-management tool is the development of a Risk-Management Plan (RMP), which summarizes product knowledge and target population data at the time of application, encompassing clinical development findings, population epidemiology, disease natural history, and identification of understudied populations who may receive the drug post-authorization. This information is crucial for assessing post-marketing adverse events and potential risks. While not all identified and potential risks need to be addressed, companies must prioritize significant risks affecting benefit–risk balance through early collaboration among clinical pharmacology, drug development, epidemiology, pharmacovigilance teams, and regulatory agencies [74].

#### 4.2.2. Risk Evaluation and Mitigation Strategy (REMS)

While the EMA mandates a product’s RMP for all applications, the U.S. Food and Drug Administration (FDA) may mandate a Risk Evaluation and Mitigation Strategy (REMS) for certain medications to ensure that benefits outweigh potential risks.

REMS is a drug safety program that the FDA requires for certain medications with serious safety concerns. While all medications have labeling that describes risks, REMS specifically focuses on preventing, monitoring and/or managing serious risks through education and reinforcement of safe medication use behaviors, rather than mitigating all adverse events [75].

REMS focuses on specific strategies that may be outlined in Part V of the RMP, which may include the following: a Medication Guide educating patients regarding safety risks and proper product usage; a Communication Plan for healthcare providers regarding safety concerns; and Elements to Assure Safe Use (ETASU), which may restrict product distribution through mechanisms such as requiring prescriber and pharmacist certification, limiting use to inpatient settings, patient monitoring, restricting dispensing to patients with documentation of safe-use conditions, or mandating enrollment in a patient registry [74]. Although the implementation of an RMP and REMS occurs in the post-approval stage, planning and study design, including the development of effective post-approval tools and risk reduction approaches, should be established during the pre-approval phase [74].

## 5. Emerging Challenges in Global Pharmaceutical Regulation

### 5.1. Advanced Therapy Medicinal Products (ATMPs)

As new and emerging drug-resistant or otherwise untreatable diseases increase, so does the need for innovative therapies, many of which are more advanced than existing options. These therapies, collectively termed Advanced Therapy Medicinal Products (ATMPs) in the European regulatory context, include gene therapies, cell therapies, and tissue-engineered products and often involve novel, complex mechanisms of action [8,76]. Because long-term effects may be delayed, uncertain, or incompletely characterized, these products pose distinct regulatory challenges in evaluating safety, efficacy, manufacturing consistency, and long-term follow-up. Ethical concerns—particularly around durable genetic modification and other potentially long-term effects—have also contributed to debate regarding their acceptance in recent years [77,78].

Recognizing the distinct challenges posed by ATMPs, global regulatory authorities have developed tailored classification systems and approval pathways [63,79]. However, differences in terminology, regulatory structures, and post-marketing requirements persist across regions, potentially delaying patient access and complicating global harmonization efforts [79,80]. International regulatory bodies such as the ICH and WHO have sought to address these gaps by promoting standardization and convergence in regulatory practices [73,81].

Major regulatory agencies have adopted distinct frameworks to govern ATMPs and similar innovative therapies, shaped by differences in regulatory maturity, healthcare infrastructure, and ethical considerations (Table 2). The terminology is not fully interchangeable across jurisdictions; for example, the U.S. Human Cells, Tissues, and Cellular and Tissue-Based Products (HCT/P) framework addresses product classification and related requirements, whereas many cell and gene therapies still proceed through Investigational New Drug (IND) and Biologics License Applications (BLA) pathways.

### 5.2. Global Health Emergencies

The International Coalition of Medicines Regulatory Authorities (ICMRA) is a voluntary coalition of leaders from medicines regulatory authorities that provides strategic direction to improve communication and enhance effective global crisis response mechanisms. Across the globe, agencies responsible for medicine regulation have established collaborative efforts through ICMRA to accelerate and strengthen processes for developing and approving COVID-19 therapeutics, including preventive vaccines and treatment options, and since 1 October 2019, leadership of ICMRA has been provided by the EMA’s Executive Director [92]. The coalition acknowledges the World Health Organization’s (WHO) international leadership in managing global health emergencies. Together, ICMRA and WHO encourage pharmaceutical manufacturers to ensure comprehensive access to clinical trial data for novel therapeutics and immunizations, regardless of whether these products receive full authorization, limited approval, emergency authorization, or are ultimately denied market access [93].

#### 5.2.1. Public Health Emergency of International Concern (PHEIC)

A Public Health Emergency of International Concern (PHEIC) is defined as an extraordinary event that constitutes a public health risk to other states through the international spread of disease and potentially requires a coordinated international response. This phenomenon implies a situation that is serious, sudden, unusual or unexpected; that carries implications for public health beyond the affected state’s national borders; and which may require immediate international action [94].

#### 5.2.2. Examples of Major Scenarios of Global Health Emergencies

- Case Type 1: Quality or safety issues with medicines on the market that affect public health across multiple countries [31]. For example, investigating widespread problems with medication that could be causing harm.
- Case Type 2: Shortage of approved medicines during crises, where previously available products become scarce or unavailable due to supply problems [31]. A clear example is a viral outbreak where antiviral treatments or vaccines are in short supply.
- Case Type 3: Urgent need for new treatments or vaccines during emerging health threats such as the COVID-19 pandemic. These situations typically arise when the WHO declares a Public Health Emergency, prompting national health authorities and the WHO to act using existing international health guidelines [31].

To improve coordination between ICMRA members and the WHO, the coalition encourages the WHO to contact ICMRA leadership when regulatory action is needed during global health emergencies [31].

### 5.3. Case Studies in Regulatory Innovation

#### COVID-19 Vaccine Development

The recent COVID-19 pandemic challenged regulatory authorities worldwide to achieve unprecedented levels of innovation. As the world grappled with the novel infectious disease, there was a pressing need for prevention and treatment options. Table 3 summarizes selected emergency or conditional authorization pathways used by several agencies for COVID-19 vaccines; the WHO Emergency Use Listing (EUL) is referenced as a distinct international listing mechanism rather than a national marketing authorization. In 2021, the FDA authorized the first COVID-19 vaccine after determining that it met the scientific standards for emergency use authorization [95,96]. The EMA used a conditional marketing authorization pathway [97,98]. The rapid emergence of COVID-19 vaccines in record time was enabled by highly adaptable platforms such as messenger RNA (mRNA) and by structural biology tools used to design immunogens that can stimulate the immune system effectively [99]. This decision reflects the principle of beneficence, which seeks to minimize risk and maximize benefits in the promotion of health and wellbeing [100].

## 6. Discussion

The comparisons presented in this review show that global pharmaceutical regulation is increasingly shaped by a tension between regulatory agility and evidentiary rigor. Established authorities such as the FDA [11], the EMA-coordinated EU regulatory network [21,22], and the PMDA [28] benefit from mature review pathways and established inspection and post-marketing infrastructures. Emerging authorities are continuing to strengthen operational capacity, digital submissions, and pharmacovigilance infrastructures while responding to rapidly growing regional demand [41,53]. At the same time, harmonization initiatives such as ICH [47] and broader international collaboration models [50] have reduced duplication and created more consistent scientific expectations across jurisdictions. However, differences in local data requirements, transparency practices, timelines, and legal mandates remain and can shape national implementation [10]. In this context, harmonization is best understood not as complete uniformity but as a pragmatic effort to align core scientific standards while preserving room for local public health priorities and regulatory capacity.

These differences become particularly consequential for complex products and crisis settings. Advanced therapies require regulators to make high-stakes decisions under uncertainty and to rely on strong lifecycle monitoring and risk management [74], particularly for Advanced Therapy Medicinal Products [79,84]. Expedited and emergency authorization pathways, illustrated most clearly during the COVID-19 pandemic [96], further amplify the need for clear post-authorization commitments [101] and active pharmacovigilance [22,71]. This review therefore suggests that regulatory effectiveness depends not only on formal statutes and review procedures but also on enabling infrastructure such as manufacturing oversight and inspection systems, prescription registries [24,27], interoperable data platforms, trained personnel, and international communication networks that support signal detection and coordinated action [92]. From a policy perspective, resilient regulation is built between crises, through investment in institutions, data governance, and work-sharing relationships, rather than during emergency authorizations alone. This is especially relevant for lower-resourced settings, where strategic reliance and regional cooperation may offer a realistic route to timely oversight without diluting accountability [41]. Because this article is a narrative comparative review focused on human medicines, it is intended to identify functional patterns across jurisdictions rather than provide a systematic inventory of every pathway or an exhaustive assessment of devices, cosmetics, or other regulated product classes.

An additional cross-cutting insight is that convergence in technical standards does not automatically translate into convergence in decision-making. Even where regulators draw on common ICH guidance [47], differences in statutory remits, benefit–risk tolerances, and requirements for local data can produce divergent evidentiary thresholds, timelines, and conditions of use [10]. Reliance and work-sharing arrangements can mitigate duplication [52], but they depend on mutual trust, transparency of assessment rationales, and clear delineation of accountability, particularly when one authority adopts another’s decision or when networks coordinate decentralized evaluations. Strengthening public-facing documentation of regulatory rationales and post-authorization commitments is therefore a central complement to harmonization efforts [93].

Digital transformation is increasingly an enabler of such coordination. Wider adoption of standardized electronic dossiers and interoperable pharmacovigilance databases can support faster signal detection and more consistent lifecycle oversight [22,71]. Digital tools are also reshaping evidence generation across the product life cycle, including the conduct and monitoring of clinical trials [66]. However, digitalization raises challenges around data governance, security, and the representativeness of real-world data sources, reinforcing the need for transparency and data integrity principles [93]. For emerging and lower-resourced authorities, targeted investments in regulatory science training and quality management systems [64] are likely to determine whether reliance mechanisms strengthen capacity and equity rather than creating long-term dependency [41].

### Strength and Limitations

A key strength of this review is the structured comparative framework anchored in primary regulator/network documentation, with table entries cross-checked against authoritative sources where feasible. However, this review is intended as a structured narrative synthesis rather than a systematic or scoping review; therefore, the literature identification approach was targeted and may not capture every relevant publication, jurisdiction-specific document, or emerging reform. In addition, regulatory systems are dynamic (guidance, organizational arrangements, and pathway names can change over time), so tabled comparisons inevitably represent a time-bounded snapshot and may not reflect subsequent updates. Comparisons are also constrained by uneven public transparency and differences in legal traditions, resourcing, and regulatory maturity across jurisdictions; accordingly, the tables simplify complex frameworks and may not fully capture within-jurisdiction variation (e.g., product-class-specific requirements, sub-national implementation, or discretionary practices). Finally, this review focuses on structures and processes rather than empirically measuring regulatory performance (e.g., review quality, timelines, inspection effectiveness, or patient outcomes), which would require dedicated datasets and analytic designs beyond the scope of this review.

## 7. Conclusions

This review shows that pharmaceutical regulation can no longer be understood as a collection of isolated national systems. It is increasingly an interconnected framework of scientific standards, institutional coordination, and lifecycle oversight intended to balance timely access to innovation with enduring expectations for safety, efficacy, and quality. Although jurisdictions differ in legal authority, resourcing, and procedural maturity, the comparative evidence across this manuscript points to a consistent lesson: strong regulation depends not only on pre-approval review but also on pharmacovigilance, inspection capacity, data infrastructure, and sustained collaboration across borders. Advanced therapies and Public Health Emergencies make these interdependencies more visible, but they do not change the core objective of regulation—to protect patients while enabling responsible innovation. Rather than pursuing uniformity for its own sake, future progress will depend on building trustworthy, transparent, and interoperable regulatory systems that can learn from one another and respond coherently to shared risks while remaining attentive to local health needs.

## 8. Future Directions

Future work in global regulatory science would benefit from four linked priorities: expanding reliance and work-sharing approaches with transparent governance; establishing fit-for-purpose standards for real-world evidence, registries, and long-term follow-up, particularly for advanced and highly individualized therapies; modernizing the digital infrastructure that supports submissions, inspections, and pharmacovigilance; and investing in regulatory capacity building through workforce development and regional partnerships. Together, these steps can help regulators accelerate access where appropriate while strengthening lifecycle oversight and maintaining scientific and ethical accountability across jurisdictions.

## Figures and Tables

**Table 1:** Comparing regulatory framework and harmonization efforts across key regulatory authorities.

Feature Category	Feature	FDA (USA) ^a^	EU Regulatory Network (EMA + NCAs) ^a^	PMDA (Japan) ^b^	NMPA (China) ^c^	ANVISA (Brazil) ^d^	CDSCO (India) ^e^	Roszdravnadzor (Russia) ^c^
General Framework	Public Health Mission	✓	✓	✓	✓	✓	✓	✓
	Legal Basis	✓	✓	✓	✓	✓	✓	✓
	Organizational Model	Team	Rapporteur	Team	Provincial	Centralized	Centralized	Federal
Pre-Market Requirements	Review Timeline (months)	6–10 ^f^	7–11 ^g^	12 ^h^	~10 ^i^	~12 ^j^	12–18 ^k^	18–26
	Expedited Pathways	Multiple	PRIME	Sakigake	Yes	Yes	Limited	Limited
	Clinical Data: Local Required	No ^l^	Partial ^m^	Yes	Yes ^n^	Partial	Yes	Yes
	Electronic Submissions (eCTD)	Yes	Yes	Yes	In progress	In progress	Partial	Paper/electronic hybrid
Post-Market Oversight	Safety Monitoring	REMS	RMP	Re-exam	Yes	Yes	Limited	Basic
	Transparency	High	Moderate	Moderate	Low	Moderate	Low	Low
	Inspection Capacity	High	High	High	Medium	Medium	Limited	Periodic
Global Alignment	ICH Participation	Full	Full	Full	Member	Observer	Limited	None
	Accept RWE (Real World-Evidence)	Growing	Developing	Developing	Limited	Limited	Minimal	Minimal

Note: Review timelines are reported as described in the cited sources for human medicines and may not reflect identical regulatory metrics across jurisdictions; they are presented for broad descriptive comparison only. Qualitative descriptors reflect the characterization in the cited literature and source materials and should be interpreted as broad comparative summaries rather than standardized quantitative scores. ✓ = feature present. “Partial” = conditional or limited requirement. “High/Moderate/Low” = degree of implementation/maturity. ^a^ Sources: [10,51]. ^b^ Source: [52]. ^c^ Source: [53]. ^d^ Sources: [51,53]. ^e^ Sources: [53,54]. ^f^ Source: [11]. ^g^ Source: [21]. ^h^ Source: [28]. ^i^ Source: [55]. ^j^ Source: [56]. ^k^ Sources: [57,58]. ^l^ Source: [59]. ^m^ Source: [60]. ^n^ Source: [61]. “EU” reflects the EU medicines regulatory network coordinated by EMA and implemented with national competent authorities (e.g., Finland’s Fimea), which perform substantial regulatory, inspection, and pharmacovigilance activities at the Member State level.

**Table 2:** Comparative overview of ATMP regulatory frameworks across major jurisdictions.

Agency/Region	Terminology	Legal Framework	Approval Pathway	Special Features	Post-Marketing Oversight
FDA (USA) ^a^	HCT/Ps, cell/gene therapies	21 CFR Part 1271	IND + BLA	RMAT, Breakthrough Fast Track	REMS + post-marketing surveillance
EU (EMA + Member States/NCAs) ^b^	Advanced Therapy Medicinal Products (ATMPs)	Regulation (EC) No. 1394/2007	Centralized EMA authorization	Hospital exemption; optional classification	RMPs required
PMDA (Japan) ^c^	Regenerative Medical Products	PMD Act (2014), Act on the Safety of Regenerative Medicine	Conditional/time-limited approval	Accelerated approval + post-market conditions	Mandatory follow-up studies
ANVISA (Brazil) ^d^	Produtos de Terapias Avançadas	RDC 338/2020	Clinical trials + GMP + submission	Pathways for rare disease therapies	Traceability + pharmacovigilance
Roszdravnadzor (Russia) ^e^	Biomedical Cell Products (BCPs)	Federal Law No. 180-FZ (2016), Law No. 61-FZ	State registration post-expert review	Centralized registry + annual reporting	Biosecurity, ethics board, and monitoring
CDSCO (India) ^f^	Stem Cell and Cell-Based Products (SCCPs)	Guidelines for Stem Cell Research (2017)	Ethics + regulatory clearance from DCGI	Institutional Ethics Committee oversight	Ethics and informed consent focus
NMPA (China) ^g^	Cell and Gene Therapy Products	Technical Guidelines (2021), ICH-aligned	IND-equivalent + GMP	Fast-track pathways	Post-marketing safety guidance

^a^ Sources: [82,83]. ^b^ Source: [84]. ^c^ Source: [85]. ^d^ Sources: [86,87]. ^e^ Source: [88]. ^f^ Source: [89]. ^g^ Sources: [90,91]. EU hospital exemption provisions and certain oversight functions are implemented at Member State level by NCAs (e.g., Finland’s Fimea), alongside EU-level coordination via EMA.

**Table 3:** Selected COVID-19 vaccine emergency or conditional authorization pathways across agencies.

Agency/Region	Pathway	Clinical Data	Post-Authorization
FDA (USA) ^a^	Emergency use authorization	Phase III trial data	RWE and active safety monitoring
EU (EMA + European Commission) ^b^	Conditional marketing authorization	Phase III data	Continued data submission
PMDA (Japan) ^c^	Special approval for emergency	Japanese or foreign Phase III trial data	Post-approval monitoring
ANVISA (Brazil) ^d^	Emergency use authorization	Phase III data	Follow-up safety/efficacy data
Roszdravnadzor (Russia) ^e^	Emergency approval	No Phase III data at initial approval	Post-approval monitoring
CDSCO (India) ^f^	Emergency use authorization	Phase II data; bridging trials may be required.	Post-approval follow-up
NMPA (China) ^g^	Conditional market approval	Phase III data	Continued research and study results

^a^ Source: [96]. ^b^ Source: [101]. ^c^ Source: [102]. ^d^ Source: [103]. ^e^ Source: [104]. ^f^ Source: [105,106]. ^g^ Source: [78].

## Data Availability

No new data were created or analyzed in this study. Data sharing is not applicable to this article.

## Supplementary Materials

The following supporting information can be downloaded at https://www.mdpi.com/article/10.3390/pharmacy14020050/s1, Table S1: Comparing regulatory framework and harmonization efforts across key regulatory authorities.

## Abbreviations

The following abbreviations are used in this manuscript:

ADR	Adverse Drug Reaction
AMA	African Medicines Agency
BLA	Biologics License Application
CHMP	Committee for Medicinal Products for Human Use
CIRS	Center for Innovation in Regulatory Science
EUL	Emergency Use Listing
HCT/Ps	Cellular and Tissue-Based Products
CFR	Code of Federal Regulations
IND	Investigational New Drug
DCGI	Drugs Controller General of India
GMP	Good Manufacturing Practice
NDA	New Drug Application
PV	Pharmacovigilance
RA	Regulatory Affairs
REMS	Risk Evaluation and Mitigation Strategy
RMP	Risk Management Plan
RMAT	Regenerative Medicine Advanced Therapy
RWE	Real-World Evidence
WHO	World Health Organization

## References

[1] Rupnawar M., Wani P. (2024). Pharmaceutical Regulatory Affairs: An Overview of Global Regulatory Frameworks and Emerging Trends. Int. J. Sci. Res. Technol..

[2] Hussaarts L., Mühlebach S., Shah V.P., McNeil S., Borchard G., Flühmann B., Weinstein V., Neervannan S., Griffiths E., Jiang W. (2017). Equivalence of Complex Drug Products: Advances in and Challenges for Current Regulatory Frameworks. Ann. N. Y. Acad. Sci..

[3] Raut N., Bajaj K., Matte P., Vhora M., Kale V., Umekar M., Harane S. (2023). Building Bridges: Harmonization Efforts for Enhanced Collaboration between Developed and Developing Countries. Int. J. Drug Regul. Aff..

[4] Darrow J.J., Avorn J., Kesselheim A.S. (2020). FDA Approval and Regulation of Pharmaceuticals, 1983–2018. JAMA.

[5] Tobin J.J., Walsh G. (2023). Medical Product Regulatory Affairs: Pharmaceuticals, Diagnostics, Medical Devices.

[6] Gherghescu I., Delgado-Charro M.B. (2021). The Biosimilar Landscape: An Overview of Regulatory Approvals by the EMA and FDA. Pharmaceutics.

[7] Muselík J., Komersová A., Kubová K., Matzick K., Skalická B. (2021). A Critical Overview of FDA and EMA Statistical Methods to Compare In Vitro Drug Dissolution Profiles of Pharmaceutical Products. Pharmaceutics.

[8] Chen R., Manochakian R., James L., Azzouqa A.-G., Shi H., Zhang Y., Zhao Y., Zhou K., Lou Y. (2020). Emerging Therapeutic Agents for Advanced Non-Small Cell Lung Cancer. J. Hematol. Oncol..

[9] Ali F., Ilyas A., Ahmadeen S. (2024). Regulations in Japan. Global Regulations of Medicinal, Pharmaceutical, and Food Products.

[10] Nagai S. (2019). Flexible and Expedited Regulatory Review Processes for Innovative Medicines and Regenerative Medical Products in the US, the EU, and Japan. Int. J. Mol. Sci..

[11] US Food and Drug Administration (FDA) (2019). Step 4: FDA Drug Review.

[12] Van Norman G.A. (2016). Drugs and Devices: Comparison of European and U.S. Approval Processes. JACC Basic Transl. Sci..

[13] Hussain F. (2013). Kefauver-Harris Drug Amendments 1962. Pharma Mirror Magazine.

[14] US Food and Drug Administration (FDA) (2018). Federal Food, Drug, and Cosmetic Act (FD&C Act).

[15] US Food and Drug Administration (FDA) (2024). What We Do.

[16] Wang W., Wertheimer I.A. (2022). History, Status, and Politicization of the FDA. Res. Soc. Adm. Pharm..

[17] Finnish Medicines Agency (Fimea) Pharmaceutical Safety and Information. https://fimea.fi/en/pharmaceutical_safety_and_information.

[18] European Medicines Agency (EMA) (2015). Adaptive Pathways.

[19] European Medicines Agency (EMA) Marketing Authorisation. https://www.ema.europa.eu/en/human-regulatory-overview/marketing-authorisation.

[20] European Medicines Agency (EMA) National Competent Authorities (Human). https://www.ema.europa.eu/en/partners-networks/eu-partners/eu-member-states/national-competent-authorities-human.

[21] European Medicines Agency (EMA) (2019). Authorization of Medicines.

[22] European Medicines Agency (EMA) Pharmacovigilance: Overview. https://www.ema.europa.eu/en/human-regulatory-overview/pharmacovigilance-overview.

[23] Finnish Medicines Agency (Fimea) Pharmacovigilance. https://fimea.fi/en/supervision/pharmacovigilance.

[24] Kanta Services (2025). Prescriptions and Medicines.

[25] Kanta Services (2025). Prescription.

[26] Swedish Medical Products Agency (Läkemedelsverket) About the Swedish Medical Products Agency. https://www.lakemedelsverket.se/en/about-the-swedish-mpa.

[27] Socialstyrelsen (National Board of Health and Welfare) (2020). National Prescribed Drug Register.

[28] Pharmaceuticals and Medical Devices Agency (PMDA). Outline of PMDA. https://www.pmda.go.jp/english/about-pmda/outline/0005.html.

[29] Pharmaceuticals and Medical Devices Agency (PMDA) History. https://www.pmda.go.jp/english/about-pmda/outline/0002.html.

[30] Wileman H., Mishra A. (2010). Drug Lag and Key Regulatory Barriers in the Emerging Markets. Perspect. Clin. Res..

[31] International Coalition of Medicines Regulatory Authorities (ICMRA) Framework for the Involvement of Health Regulatory Authorities in the Management of Global Health Crises. https://www.icmra.info/drupal/sites/default/files/2019-04/ICMRA%20SOP%20for%20Crisis%20Management.pdf.

[32] Ferreira C.G. (2019). ES10.05 Brazilian Health Regulatory Agency—Agência Nacional de Vigilância Sanitária. J. Thorac. Oncol..

[33] Huynh-Ba K., Sassi A.B. (2018). ANVISA: An Introduction to a New Regulatory Agency with Many Challenges. AAPS Open.

[34] Dhole S.D., Darade B.R. (2024). Indian Pharmaceutical Regulatory Authority: Review. Int. J. Pharm. Sci..

[35] Central Drugs Standard Control Organization (CDSCO) (2025). Introduction.

[36] Federal Service for Surveillance in Healthcare Regulatory Framework for Federal Service for Surveillance in Healthcare. https://roszdravnadzor.gov.ru/en.

[37] Vargas-Pelaez C.M., Drago M.T., Acosta A., Farias M.R. (2017). Pharmaceutical Policy in Argentina. Pharmaceutical Policy in Countries with Developing Healthcare Systems.

[38] Argentina.gob.ar What Is ANMAT? 2025. https://www.argentina.gob.ar/anmat/anmat-en/what-anmat.

[39] Skerritt J. (2022). Australia’s Robust Strategy for Regional and Global Medical Product Engagement.

[40] African Union Development Agency–New Partnership for Africa’s Development (AUDA-NEPAD). Who We Are. African Medicines Regulatory Harmonization (AMRH). https://amrh.nepad.org/amrh-microsite/who-we-are.

[41] World Health Organization (WHO) (2024). Senegal and Rwanda Achieve WHO Maturity Level 3 in Medicines Regulation.

[42] Ozili P.K. (2021). COVID-19 Pandemic and Economic Crisis: The Nigerian Experience and Structural Causes. J. Econ. Adm. Sci..

[43] Olatunji O.E. (2014). Leadership Effectiveness and Regulatory Performance in the Public Sector: The Experience of the National Agency for Food and Drug Administration and Control (NAFDAC). Int. J. Innov. Res. Dev..

[44] Omijeh B.O., Raheem O. (2021). An Improved Drug Verification System for NAFDAC. Afr. Sch. J. Afr. Sustain. Dev..

[45] Spink J., DCMoyer Rip M.R. (2016). Addressing the Risk of Product Fraud: A Case Study of the Nigerian Combating Counterfeiting and Sub-standard Medicines Initiatives. J. Forensic Sci. Criminol..

[46] National Agency for Food and Drug Administration and Control (NAFDAC) About NAFDAC. https://nafdac.gov.ng/about-nafdac/.

[47] International Council for Harmonisation (ICH) History. Official Web Site: ICH. https://www.ich.org/page/history.

[48] Adleberg J. (2024). ICH Overview, Proceedings of the FDA/HC ICH Regional Public Meeting, Virtual, 22 February 2024.

[49] International Council for Harmonisation (ICH) Management Committee. https://www.ich.org/page/management-committee.

[50] Reggi V. (2017). Medicines Regulatory Harmonization: International Collaboration as a Key to Improve Public Health. Med. Access Point Care.

[51] Eurofins Scientific Three Key Regulations in the Pharmaceutical Industry: EMA, FDA and ANVISA. https://www.eurofins.com/assurance/resources/articles/three-key-regulations-in-the-pharmaceutical-industry-ema-fda-and-anvisa/.

[52] Center for Innovation in Regulatory Science (CIRS) (2021). New Drug Approvals in Six Major Authorities 2011–2020: Focus on Facilitated Regulatory Pathways and Worksharing.

[53] Vashisth S., Singh G., Nanda A. (2012). A Comparative Study of Regulatory Trends of Pharmaceuticals in Brazil, Russia, India and China (BRIC) Countries. J. Generic Med..

[54] Clini India (2024). Understanding US FDA vs. EMA vs. CDSCO: How Do They Differ?.

[55] National Medical Products Administration (NMPA) (2025). Measures Facilitate Approval of 48 First-in-Class Innovative Drugs.

[56] Agência Nacional de Vigilância Sanitária (Anvisa) Drugs. https://www.gov.br/anvisa/pt-br/english/regulation-of-products/drugs.

[57] Deepika M., Karthi J. (2025). A Comparative Study of USFDA, EMA, and CDSCO Regulatory Guidelines. Int. J. Res. Publ. Rev..

[58] Konwar M., Maurya M.R., Nishandar T.B., Thatte U.M., Gogtay N.J. (2021). An evaluation of drug lag for new drugs approved by the Indian regulator relative to the United States, European Union, and Japanese regulatory agencies: A 15-year analysis (2004–2018). Perspect. Clin. Res..

[59] US Food and Drug Administration (FDA) (2025). FDA Acceptance of Foreign Clinical Studies Not Conducted Under an IND: Frequently Asked Questions.

[60] Naito C. (1998). Ethnic Factors in the Acceptability of Foreign Clinical Data. Drug Inf. J..

[61] National Medical Products Administration (NMPA) (2020). Clinical Technical Requirements for Drugs Marketed Overseas but Not Marketed in China.

[62] US Department of Health and Human Services (2018). Read the Belmont Report.

[63] Abou-El-Enein M., Elsanhoury A., Reinke P. (2016). Overcoming Challenges Facing Advanced Therapies in the EU Market. Cell Stem Cell.

[64] International Council for Harmonisation (ICH) (2016). Guideline for Good Clinical Practice E6(R2).

[65] Council for International Organizations of Medical Sciences, World Health Organization (2002). International Ethical Guidelines for Biomedical Research Involving Human Subjects.

[66] Rosa C., Marsch L.A., Winstanley E.L., Brunner M., Campbell A.N.C. (2021). Using Digital Technologies in Clinical Trials: Current and Future Applications. Contemp. Clin. Trials.

[67] US Food and Drug Administration (FDA) (2025). CDER Center for Clinical Trial Innovation (C3TI).

[68] Scheppler L., De Clercq N., McGoldrick M., Dias J. (2021). Regulatory Harmonization and Streamlining of Clinical Trial Applications Globally Should Lead to Faster Clinical Development and Earlier Access to Life-Saving Vaccines. Vaccine.

[69] Hwenda L., Sidibe M., Makanga M. (2022). The African Medicines Agency: The Key to Unlocking Clinical Research in Africa. Lancet Glob. Health.

[70] Jackson C. (2020). Africa’s Missing Genomic Data and Its Impact on Health Care. GEN—Genetic Engineering and Biotechnology News.

[71] Khan M.A.A., Hamid S., Babar Z.U.D. (2023). Pharmacovigilance in High-Income Countries: Current Developments and a Review of Literature. Pharmacy.

[72] Cui X., Wang L.X., Liu G.Y., Xie Y.M. (2021). Enlightenment of International Pharmacovigilance System on Establishment of Pharmacovigilance System of Chinese Medicine. China J. Chin. Mater. Medica.

[73] World Health Organization (WHO) (2020). 72nd and 73rd Report: WHO Technical Report Series No. 1030.

[74] Dieck G.S., Sharrar G.R. (2013). Preparing for Safety Issues Following Drug Approval: Pre-Approval Risk Management Considerations. Ther. Adv. Drug Saf..

[75] US Food and Drug Administration (FDA) (2023). Risk Evaluation and Mitigation Strategies (REMS).

[76] Allison S.J. (2022). Novel Anti-Cancer Agents and Cellular Targets and Their Mechanism(s) of Action. Biomedicines.

[77] Rothschild J. (2020). Ethical Considerations of Gene Editing and Genetic Selection. J. Gen. Fam. Med..

[78] (2020). China Grants Conditional Approval for First COVID Vaccine.

[79] Hanna E., Rémuzat C., Auquier P., Toumi M. (2016). Advanced Therapy Medicinal Products: Current and Future Perspectives. J. Mark. Access Health Policy.

[80] Cuende N., Rasko J.E.J., Koh M.B.C., Dominici M., Ikonomou L. (2018). Cell, Tissue and Gene Products with Marketing Authorization in 2018 Worldwide. Cytotherapy.

[81] International Council for Harmonisation (ICH) Mission. https://www.ich.org/page/mission.

[82] US Food and Drug Administration (FDA) Regulation of Human Cells, Tissues, and Cellular and Tissue-Based Products (HCT/Ps). https://www.fda.gov/vaccines-blood-biologics/cellular-gene-therapy-products.

[83] US Food and Drug Administration (FDA) Regenerative Medicine Advanced Therapy (RMAT) Designation. https://www.fda.gov/vaccines-blood-biologics/cellular-gene-therapy-products/regenerative-medicine-advanced-therapy-designation.

[84] European Medicines Agency (EMA) (2024). Advanced Therapy Medicinal Products: Overview.

[85] Pharmaceuticals and Medical Devices Agency (PMDA) Review of Regenerative Medical Products. https://www.pmda.go.jp/english/review-services/reviews/0003.html.

[86] National Health Surveillance Agency—Anvisa (2020). 10154json-file-1.pdf. https://www.gov.br/anvisa/pt-br/assuntos/reunioesdadicol/processos-deliberados/5de2020/arquivos/10154json-file-1/view.

[87] Brazilian Health Regulatory Agency (Anvisa) (2023). Anvisa Approves Specific Good Manufacturing Practice Regulation for Advanced Therapy Products.

[88] Roszdravnadzor Federal Law No. 180-FZ on Biomedical Cell Products. https://www.consultant.ru/document/cons_doc_LAW_200349/.

[89] Jotwani G. (2017). National Guidelines for Stem Cell Research.

[90] Yin C., Gao J., Li G., Hu H., Zhou L., Lu S., Chen X. (2022). Gene and Cell Therapies in China: Booming Landscape under Dual-Track Regulation. J. Hematol. Oncol..

[91] Li Y., Verter F., Wang B., Gu N. (2019). Regulations on Cell Therapy Products in China: A Brief History and Current Status. Regen. Med..

[92] International Coalition of Medicines Regulatory Authorities (ICMRA) (2016). International Coalition of Medicines Regulatory Authorities (ICMRA).

[93] World Health Organization (WHO) (2021). Joint Statement on Transparency and Data Integrity: International Coalition of Medicines Regulatory Authorities (ICMRA) and WHO.

[94] World Health Organization (WHO) (2019). Emergencies: International Health Regulations and Emergency Committees.

[95] Tanne J.H. (2021). COVID-19: FDA Approves Pfizer-BioNTech Vaccine in Record Time. BMJ.

[96] US Food and Drug Administration (FDA) (2021). FDA Approves First COVID-19 Vaccine.

[97] European Medicines Agency (EMA) (2020). EMA Recommends First COVID-19 Vaccine for Authorisation in the EU.

[98] European Centre for Disease Prevention and Control (ECDC) (2020). First COVID-19 Vaccine Authorised for Use in the European Union.

[99] Fauci A.S. (2021). The Story Behind COVID-19 Vaccines. Science.

[100] Brear M.R., Gordon R. (2021). Translating the Principle of Beneficence into Ethical Participatory Development Research Practice. J. Int. Dev..

[101] European Medicines Agency (EMA) (2016). Conditional Marketing Authorisation.

[102] Pharmaceuticals and Medical Devices Agency (PMDA) (2021). Special Approval for Emergency on First COVID-19 Vaccine in Japan.

[103] Mattos Filho (2020). Anvisa Issues Emergency Use Authorization Rules to Accelerate Access to COVID-19 Vaccines.

[104] Mikule E., Reissaar T., Villers J., Penka A.S.T., Temerev A., Rozanova L. (2021). The Fast Approval and Slow Rollout of Sputnik V: Why Is Russia’s Vaccine Rollout Slower than That of Other Nations?. Epidemiologia.

[105] Central Drugs Standard Control Organization (CDSCO) (2022). Approved COVID-19 Vaccines as on 08.07.2022. Government of India. https://cdsco.gov.in/opencms/resources/UploadCDSCOWeb/2018/UploadPublic_NoticesFiles/Approved%20COVID-19%20vaccines%20as%20on%2008.07.2022.pdf.

[106] Central Drugs Standard Control Organization (CDSCO) (2021). Recommendations of the SEC Meeting to Examine COVID-19 Related Proposals Under Accelerated Approval Process Made in Its 134th Meeting Held on 01.01.2021. Government of India. https://cdsco.gov.in/opencms/resources/UploadCDSCOWeb/2018/UploadCommitteeFiles/134th%20COVID-19%20Recommendation%2001.01.2021.pdf.

