# Supplementary material for: Global Pharmaceutical Regulation: Comparative Frameworks and Operations
Source: Pharmacy (Basel). 2026 Mar 18;14(2):50. doi: 10.3390/pharmacy14020050 (PMC13010624; doi:10.3390/pharmacy14020050)
Supplement: Supplementary file 1 [file pharmacy-14-00050-s001.zip › Table S1.pdf]

Table S1. Comparing Regulatory Framework and Harmonization Efforts Across Key Regulatory Authorities.

Feature	FDA (US)	EU regulatory network (EMA + NCAs)	PMDA (Japan)	NMPA (China)	ANVISA (Brazil)	CDSCO (India)	Roszdravnadzor (Russia)	Similar(S) or Different (D)
**Core Mission**	Protection of public health through drug safety, efficacy, and quality assurance	Safeguarding health through scientific evaluation of medicines	Protection of public health through ensuring safety, efficacy, and quality	Regulating safety, quality, and efficacy of drugs and medical devices	Managing risks of health-related goods and services	Safeguarding public health through product safety, efficacy, and quality	Ensuring safety and quality of medicines and medical devices	**S - All share public health protection mission**
**Organizational Structure**	Team-based review with Advisory Committees	Rapporteur/Co-rapporteur with Scientific Committees	Team-based evaluation with consultation system	Provincial review with central authority	Centralized within Brazilian Health System	Central authority with state coordination	Federal executive body under Ministry	**D - Each has distinct structure**
**Standard Review Timeline**	6-10 months	7-11 months	12 months	200 working days	365 days	12-18 months	18-26 months	**SD - All have defined timelines, although different in duration**
**Legal Framework**	Federal Food, Drug, and Cosmetic Act with amendments	EU Pharmaceutical legislation	Pharmaceuticals and Medical Devices Act	Drug Administration Law	Health Surveillance Law	Drugs and Cosmetics Act	Federal healthcare legislation	**S - All have specific legislative basis**
**Expedited Programs**	Multiple (Fast Track, Breakthrough, Accelerated, Priority)	PRIME, Accelerated Assessment, Conditional Marketing Authorization	Sakigake designation	Special approval pathway	Simplified procedure for priority products	Accelerated approval for specific conditions	Expedited review for life-saving drugs	**SD - All have some form, Although different in scope and criteria**

**Clinical Data Requirements**	Accepts international data	Accepts international data (requires EU-based patient data)	Requires Japanese patient data	Requires Chinese patient data	Accepts international data with local requirements	Requires Indian patient data	Preference for local data	**D - Varying requirements for local representation**
**Quality Standards**	cGMP requirements, Quality by Design approach	EU GMP standards, detailed quality expectations	ICH-based requirements with local specifications	Chinese Pharmacopeia requirements	Brazil-specific GMP standards	Indian Pharmacopeia standards	Russian Federation standards	**SD - All require quality standards, Although different in specific requirements**
**Post-market Safety Monitoring**	REMS for high-risk products, periodic reports	RMP required for all products	Re-examination system	Post-approval safety studies	Pharmacovigilance program with periodic reports	Limited post-marketing surveillance	Post-registration monitoring system	**SD - All require some monitoring, though with difference in rigor**
**Transparency Level**	High (complete reviews published)	Moderate (EPAR summaries)	Moderate (limited information)	Low (limited public access)	Moderate (some decision rationales)	Low (limited public information)	Low (minimal public disclosure)	**D - Significant variation in transparency**
**Advisory Committee Role**	Significant role in decision-making	Scientific advice through committees	Limited role in evaluation	Expert panels with limited influence	Technical chambers provide expertise	Expert committees with decision influence	Expert councils with limited input	**D - Varies from central to peripheral**
**Harmonization Participation**	ICH founding member, active	ICH founding member, active	ICH founding member, active	ICH member since 2017	ICH Observer, The Pan American Network for Drug Regulatory Harmonization (PANDRH) p member	Limited ICH participation	Limited international involvement	**SD - Most participate in harmonization, however there is difference in degree of participation across countries.**

**Risk Management Approach**	Risk-based for selected products (REMS)	Mandatory RMP for all products	Similar to EMA approach	Developing approach	Similar to EU framework	Developing system	Limited risk management framework	**SD- All incorporate risk management, but comes with difference in implementation**
**Electronic Submission Format**	eCTD required	eCTD required	eCTD required	eCTD implementation in progress	eCTD implementation in progress	Paper and electronic hybrid	Paper-based with electronic elements	**SD - Moving toward electronic, but countries are at different implementation stage**
**Market and Data Exclusivity Period**	5 years (New Chemical Entity, NCE), 3 years (new indications)	8+2+1 year system (8 years of data exclusivity, 2 years of market protection, +1 potential extension)	8 years	6 years	No official data exclusivity period fully implemented.	No official data exclusivity; relies on patent law for protection	Limited protection, with variable exclusivity terms	**D - Significant variation in protection periods**
**Biosimilar Pathway**	351(k) pathway with extensive guidelines	Well-established pathway with detailed guidelines	Biosimilar guidelines similar to EMA	Developing pathway	Developing pathway	Abbreviated pathway	Limited framework	**S - All have an existing framework, but at different stages of maturity**
**RWE Acceptance**	Growing acceptance with formal program	Limited acceptance with guidelines developing	Limited acceptance	Very limited	Very limited	Very limited	Minimal	**D - Significant variation in acceptance**
**Inspection Approach**	Risk-based with regular site visits	Coordination with national authorities	Regular inspection program	Intensive inspection requirements	National inspection program	Limited inspection capacity	Periodic inspection framework	**SD - All conduct inspections, but there are differences in frequency and approach**

**Patient Involvement**	Growing patient engagement	Formal patient representative system	Limited patient involvement	Minimal patient involvement	Growing patient engagement	Minimal patient involvement	Minimal patient involvement	**D - Significant variation in patient role**
**Fee Structure**	User fee program funds significant portion	Fee for service with member state contributions	User fee system	Fee-based system	Fee-based system	Fee-based system	State-funded with fees	**SD - All utilize fees, though there are different funding models across countries**
**Emergency Use Provisions**	Emergency Use Authorization	Conditional approval in public emergency	Special approval in emergencies	Special approval process	Emergency use provisions	Emergency use provisions	State emergency provisions	**SD - All have emergency mechanisms, although with difference in implementation**

Key/Legend:

Similar (S): The regulatory feature exists across all or most authorities with similar core principles, though implementation details may vary

Difference (D): The feature shows significant variation in approach, requirements, or implementation across authorities
